# A rapid urinary test for combining PSA and zinc to enhance prostate cancer diagnosis: results from a prospective study

**DOI:** 10.1038/s41391-025-01030-2

**Published:** 2025-12-08

**Authors:** Daniele Amparore, Sabrina De Cillis, Davide Zamengo, Michele Ortenzi, Eugenio Alladio, Fabio Di Nardo, Thea Serra, Sergio Occhipinti, Cristian Fiori, Francesco Porpiglia

**Affiliations:** 1 Department of Oncology, University of Turin, Orbassano, Turin, Italy; 2 Division of Urology, Department of Surgery, Candiolo Cancer Institute, FPO-IRCCS, Candiolo (TO), Italy; 3 Division of Urology, San Luigi Hospital, Orbassano, Italy; 4 Department of Chemistry, University of Turin, Turin, Italy; 5 Department of Molecular Biotechnology and Health Sciences, University of Turin, Turin, Italy

**Keywords:** Diagnostic markers, Cancer screening, Diagnostic markers

## Abstract

**Background:**

The need for simple, non-invasive biomarkers for prostate cancer (PCa) diagnosis is increasing. Urinary PSA (uPSA) and Zinc (uZinc) are emerging as potential PCa risk indicators. This study aimed to develop a rapid urine test combining uPSA and uZinc, assessing its diagnostic value alone and with Standard of Care (SOC) parameters, including age, serum PSA, DRE, and multiparametric Magnetic Resonance Imaging (mpMRI).

**Methods:**

We enrolled 260 men undergoing prostate biopsy. Post-massage urine samples were analyzed using a rapid urine test combining lateral flow immunoassay (uPSA) and colorimetric dipstick assay (uZinc) with confirmatory testing via ELISA and colorimetric in vitro assay. Logistic regression models (SOC, uPSA, uZinc, Urine test [uPSA + uZinc], SOC + Urine test) and mpMRI models were tested. Diagnostic accuracy was evaluated using AUCs from ROC analysis. A decision-making algorithm targeting patients with increased PSA up to 10 ng/mL, negative DRE, and PIRADS ≤ 3 was proposed to assess the number of unnecessary biopsies potentially avoided with the urine test.

**Results:**

Among 242 evaluable patients, 146 (59%) had PCa. The rapid Urine test provided intensity scores inversely proportional to biomarker concentration. uPSA strongly correlated with clinical stage, D’Amico risk, and Gleason score, while uZinc showed a weaker trend. The Urine test reached an AUC of 0.769, which improved performance to 0.789 with SOC + Urine test (p = 0.0002). Combining urine markers with mpMRI yielded AUCs of 0.868 (mpMRI+Urine test) and 0.875 (mpMRI+SOC+Urine test; p < 0.0001). The decision-making algorithm integrating urine test demonstrated that 51% of men could safely avoid biopsy, with a 13% detection rate of only low-grade PCas (ISUP < 2) in this group.

**Conclusions:**

This uPSA/uZinc urine test is a promising adjunct to current diagnostic pathways, improving accuracy in detecting clinically significant PCa while reducing unnecessary biopsies. Its integration with mpMRI and SOC parameters could refine risk assessment and personalize patient management.

## Introduction

Prostate cancer (PCa) is the second most common cancer in men globally [1, 2]. When a clinically significant PCa (csPCa) is detected earlier stages, it can often be treated effectively with a radical intent by surgery or radiation therapy. Advanced cases, however, are harder to treat and involve more side effects [3–6]. The development of PSA-based screening combined with digital rectal examination (DRE) of the past decades has led to decline in disease mortality [7–9]. However, PSA lacks specificity, often leading to overdiagnosis of indolent cancers and unnecessary treatments, resulting in higher healthcare costs without improving survival outcomes [10–12].

Multiparametric MRI (mpMRI) has improved diagnostic accuracy by identifying csPCa cases more reliably [13–15], reducing unnecessary biopsies, and being recently endorsed by European Association of Urology (EAU) guidelines [7]. However, mpMRI remains costly, dependent on skilled radiologists, and still faces concerns about overdiagnosis [16, 17].

To overcome these issues, several blood and urine biomarkers have been developed in the last years to improve PCa risk stratification, such as PHI, 4Kscore, SelectMDx, and PCA3, but none have gained widespread use due to limited evidence of their added benefit over standard tools [18–21]. However, the potential advantages of the urinary biomarkers in terms of sample collection, being the prostate in direct connection to the urinary tract, are pushing the research efforts in this direction. One of such marker is urinary zinc (uZinc). The prostate typically accumulates zinc for citrate storage essential for sperm function [22]. Cancer disrupts zinc uptake, lowering both tissue and urinary zinc levels, with studies showing uZinc levels decrease in PCa patients, correlating with more advanced disease [23, 24].

Similarly, loss of PSA expression in aggressive tumors, despite high serum PSA, highlights the limitations of PSA as a standalone marker [25]. Recent studies combining uZinc and uPSA have found both markers reduced in PCa patients, with correlations to Gleason Score and PIRADS [26].

These findings support their potential diagnostic value, though current testing is complex and costly [27]. Moreover, the combined analysis of uZinc and uPSA, reflecting two distinct physiological processes of the prostate, provides complementary information that may help distinguish neoplastic transformation from other benign prostatic conditions.

Based on these assumptions, the current study aims to develop a user-friendly, commercially available rapid urine test combining uZinc and uPSA for clinically significant PCa detection.

## Material and methods

### Study design and population

The study followed the Declaration of Helsinki and was approved by the Ethics Committee of San Luigi Gonzaga Hospital (Prot. No. 6387). Written informed consent was obtained from all participants, and samples were anonymized before analysis.

Men referred for prostate biopsy at San Luigi Hospital (Orbassano, Turin, Italy) were invited to join. Biopsy indications followed EAU guidelines [7], considering age, PSA, DRE, and mpMRI findings.

Inclusion criteria were age under 75, PSA > 4 ng/mL with negative DRE, suspicious DRE or suspicious mpMRI. Exclusion criteria were prior prostate biopsy in the last 6 months or previous PCa diagnosis.

### Urine sample collection, processing and analysis

Voided urine samples (45 mL) were collected following a standardized prostatic massage (three digital compressions per lobe, from base to apex, over 30 s). Biopsies were performed under transrectal ultrasound (TRUS) guidance, including both systematic and mpMRI/US fusion-targeted cores.Both transrectal and transperineal were included as biopsy approaches [28].

Pre-biopsy urinary samples were tested for uPSA and uZinc, as previously described [26].

### Lateral flow biosensor and uPSA detection

A custom competitive lateral flow biosensor was developed to detect uPSA, as previously published [29], where it was determined that at 90% sensitivity the threshold value for uPSA is 2.800 ng/ml. (Supplementary Fig. 1a). For testing, 70 μL of urine was applied to the sample pad. In the presence of PSA, antibody binding to the immobilized PSA was progressively inhibited, with complete inhibition occurring when PSA levels exceeded the cutoff. Results were visually interpreted after 10 min based on the presence or absence of the test line.

### Colorimetric strip assay fabrication and uZinc detection

For uZinc, a previous study identified at 90% of sensitivity the threshold value of 1.400 ng/ml (Supplementary Fig. 1b) [24].

Membrane filter Cobetter was saturated with Dithizone solution prepared in EtOH. Once dry, the membrane was cut into squares of ~25 mm^2^ and the test was conducted by dispensing 10 μL of patient urine. The result was assessed after 5 min with the naked eye as color change from light orange to dark fuchsia.

### Intensity scale for lateral flow and colorimetric tests

**Fig. 1:**
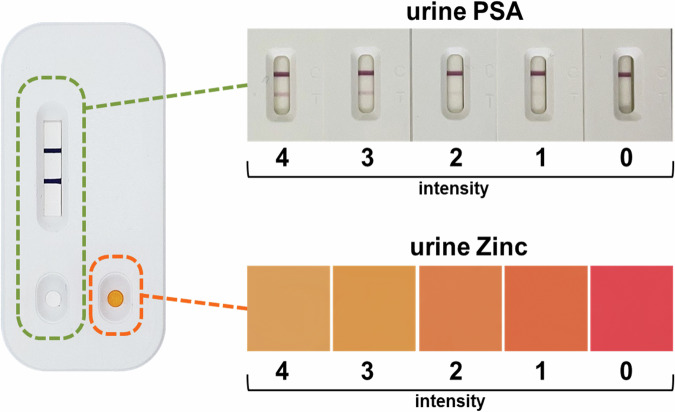
Intensity scales of the urinary PSA and urinary Zinc in the rapid combined test.

An intensity scale from values 0 to 4 was created for both uPSA and uZinc tests independently (Fig. 1), based on their correlation to the presence and aggressiveness of PCa [26]. 0–1 indicate values above cut-offs, while 2–4 reflect decreasing of each marker level. uPSA is assessed via test line intensity while uZinc by color shift from fuchsia to light orange. Combining the resulting values together it is possible to define three diagnostic scenarios (Urine score): both markers below cut-offs (score 2), one below and one above (score 1), or both above (score 0), helping stratify risk based on urinary biomarker profiles.

### Prostate biopsy decision-making algorithm development

To assess the clinical utility of a urinary biomarker test for prostate cancer (PCa), a decision-making algorithm was developed for patients with inconclusive clinical findings (PSA 4–10 ng/mL, negative DRE, and either negative mpMRI or PIRADS 3 lesion). This subset of patients reflects a setting of unclear and continuously evolving indications, as also demonstrated by the 2025 EAU Guidelines for PCa [30]. For these patients, the urine test is used to stratify risk: a score of 2 (indicating both biomarkers are positive) leads to biopsy, while scores of 0 or 1 suggest low probability of csPCa, and patients are managed with clinical follow-up, reserving biopsy for cases with persistent or evolving suspicion (Supplementary Fig. 2).

### Statistical analysis

Initial analyses compared uPSA, uZinc, and standard clinical parameters (serum PSA, age, risk factors, DRE, and mpMRI) between healthy individuals and PCa patients. Pearson’s correlation explored associations between biomarkers, clinical variables, and Gleason Score (GS).

Univariate and multivariate logistic regression models assessed the diagnostic accuracy of uPSA and uZinc, with model performance evaluated by area under the ROC curve (AUC).

Three initial models were developed: (1) the Standard of Care (SOC) model (serum PSA, age, DRE), (2) the Urine test (uPSA + uZinc), and (3) the SOC + Urine test. Additional models incorporated mpMRI (PiRADS score), resulting in four more combinations: mpMRI, mpMRI + SOC, mpMRI + Urine, and mpMRI + SOC + Urine.

AUC comparisons were done using DeLong’s test, and internal validation was performed with 1000 bootstrap iterations. Analyses used MedCalc® Statistical Software v19.8.

Decision Curve Analysis (DCA) was used to assess the clinical utility of different predictive models by measuring their net benefit across various threshold probabilities. The net benefit, plotted on the y-axis, reflects the trade-off between unnecessary interventions and missed diagnoses. Analyses were performed using the *dcurves* package in R (v4.4).

## Results

Of the 260 men initially screened for eligibility, 18 were excluded due to issues with urine sample collection. A total of 242 participants were ultimately included in the study (Table 1).

**Table 1:** Patients’ characteristics.

Patients characteristics, n	260
Evaluable samples, n (%)	242 (93)
Age, years, mean (median; IQR^1^)	69 (70; 64–75)
PSA, ng/mL, mean (median; IQR)	11 (7; 5.2–10.3)
Abnormal DRE, n (%)	76 (31)
PCa diagnosis, n (%)	144 (59)
ISUP 1, n (%)	10 (6.9)
ISUP 2, n (%)	59 (40.7)
ISUP 3, n (%)	38 (26.2)
ISUP 4, n (%)	20 (13.8)
ISUP 5, n (%)	19 (13.1)

*PSA* prostate specific antigen, *DRE* digital rectal examination, *PCa* prostate cancer, *GS* Gleason score, *IQR* interquartile range.

The mean age of the cohort was 69 years. Median serum PSA was 7 ng/mL, with an interquartile range (IQR) of 5.2 to 10.3 ng/mL. An abnormal DRE was reported in ~31% of participants.

Among the 242 men, 96 (40%) had negative biopsy results and were classified as healthy, while 146 (60%) were diagnosed with PCa. Looking at the PCa patients stratified according to the International Society of Uro-Pathology score (ISUP), 10 (6.9%) had ISUP Grade 1, 59 (40.7%) were ISUP 2, 38 (26.2%) ISUP 3, 20 (13.8%) ISUP 4, and 19 (13.1%) ISUP 5.

To assess analytical performance, all urine samples were tested within hours of collection to preserve biomarker stability. As shown in Supplementary Fig. 3, rapid test results strongly correlated with reference methods: an inverse correlation was observed between lateral flow assay and ELISA for uPSA (R = −0.64, p < 0.0001), and between the colorimetric test and mass spectrometry for uZinc (R = −0.69, p < 0.0001).

Rapid test signal intensities for uPSA and uZinc also correlated with key clinical parameters, including PCa stage, ISUP, and D’Amico risk classification, supporting their diagnostic potential.

Higher signal intensities on the uPSA lateral flow device, corresponding to lower uPSA concentrations, were consistently associated with more advanced clinical stages, risk D’Amico categories, and elevated ISUP (R = 0.43; R = 0.38; R = 0.43; p < 0.0001). A clear inverse relationship emerged between uPSA levels and PCa severity. Similarly, uZinc levels demonstrated a comparable pattern (R = 0.32; R = 0.33; R = 0.235; p < 0.0001) although the correlation was slightly weaker than what had been observed for uPSA. Moreover, both uPSA and uZinc displayed higher correlation with clinical parameters compared to the standard blood PSA (Supplementary Table 1).

**Fig. 2:**
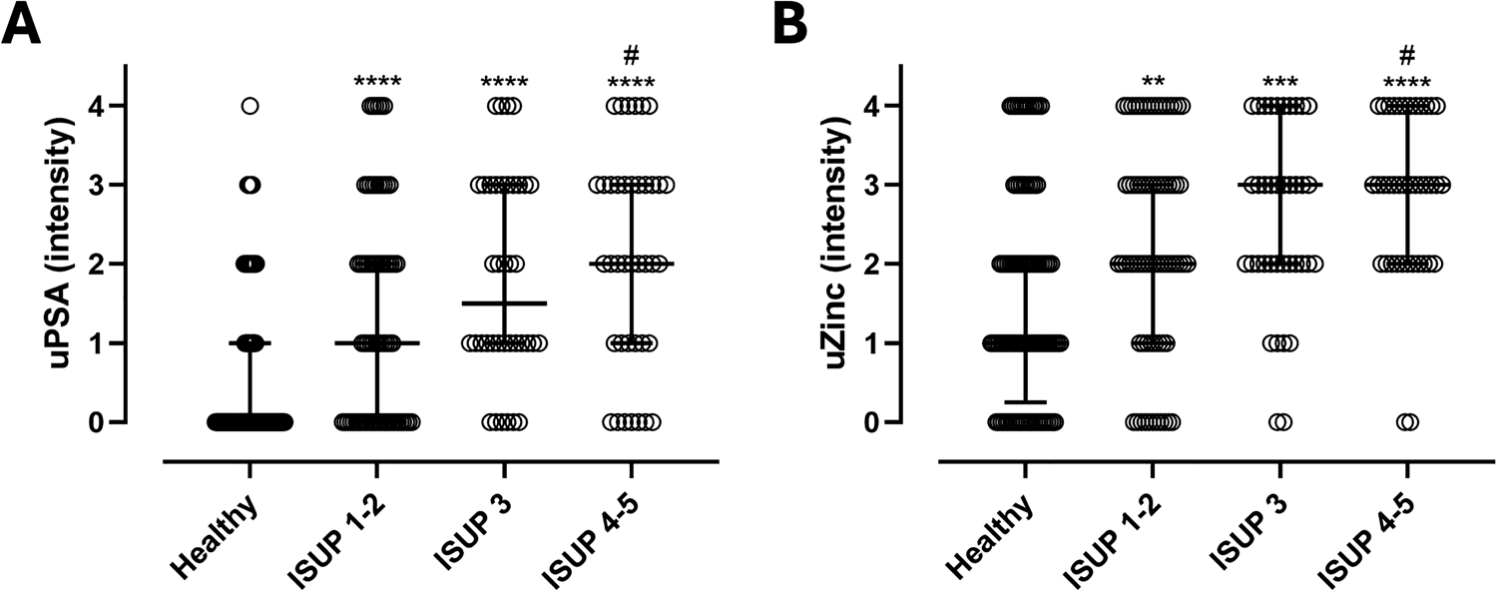
uPSA and uZinc intensity in healthy individuals and PCa patients. **A** Intensity of uPSA in healthy individuals, ISUP 1–2, ISUP 3 and ISUP 4–5 patients. **B** Intensity of uZinc in healthy individuals, ISUP 1–2, ISUP 3 and ISUP 4–5 patients. Data are presented as median with interquartile range. Statistical significance is indicated as follows: *p < 0.05, **p < 0.01, ***p < 0.001, ****p < 0.0001 compared to healthy subjects; #p < 0.05 compared to ISUP GG 1–2.

Subjects were therefore stratified into four diagnostic categories to assess the correlation between test signal intensity and PCa severity: healthy individuals (negative biopsy), ISUP 1–2, ISUP 3, and ISUP 4–5 (Fig. 2).

For urinary PSA (uPSA), intensity values were significantly higher in all patient groups compared to healthy controls (p < 0.0001). Among patients, those with ISUP grade 4–5 exhibited significantly higher intensity values than those with ISUP grade 1–2. A gradual increase in intensity was observed with increasing Gleason Grade Group, showing a significant trend (p for trend < 0.0001) (Fig. 2A).

Regarding urinary Zinc (uZinc), intensity values were progressively higher in patients with ISUP 1–2, ISUP 3, and ISUP 4–5 compared to healthy controls (p < 0.01, p < 0.001, and p < 0.0001, respectively). Moreover, ISUP 4–5 patients showed significantly higher intensity values than ISUP 1–2 patients (p < 0.05). A significant increasing trend in intensity was also observed with higher Gleason Grade Groups (p for trend < 0.0001) (Fig. 2B).

Diagnostic performance of the rapid urine test in identifying PCa was assessed using five logistic regression models, including a SOC model (based on sPSA, age, and DRE), models based on uPSA and uZinc individually, the combined Urine test (uPSA + uZinc) and a full model integrating SOC and urinary biomarkers (SOC + Urine test).

Univariate binary logistic regression analysis was performed to evaluate the individual contribution of each variable to prostate cancer detection. The results showed that all variables were positively associated with cancer risk. Specifically, the odds ratio (OR) for DRE was 1.63, for serum PSA 1.03, and for age 1.04. Among the urinary biomarkers, uPSA showed a strong association with an OR of 2.19, while uZinc had an OR of 1.41. The highest predictive value was observed for the PIRADS score, with an OR of 3.57 (Supplementary Table 2).

**Fig. 3:**
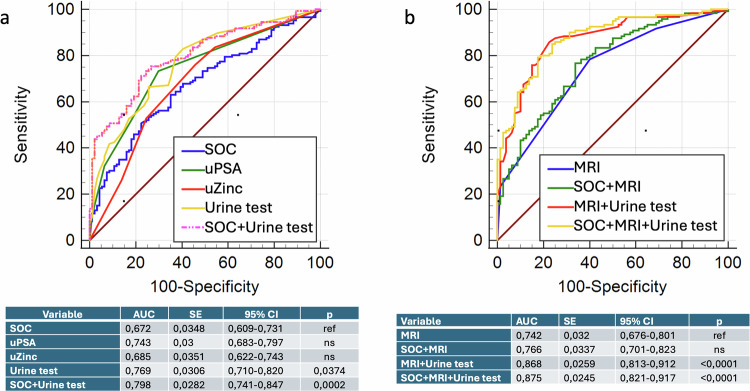
ROC curve analysis of the diagnostic performance of the rapid urine test. **a** Comparison of five logistic regression models: SOC, uPSA alone, uZinc alone, uPSA + uZinc, and SOC + urinary biomarkers. **b** Comparison of four logistic regression models in patients who underwent mpMRI (n = 200): mpMRI alone, mpMRI + Urine test (uPSA + uZinc), MRI + SOC, and mpMRI + SOC + Urine test.

ROC curve analysis showed that the model based on urinary biomarkers Urine test achieved an AUC of 0.769, while the integration of these markers with SOC increased the AUC to 0.789. This improvement was statistically significant compared to SOC alone (p = 0.0002) (Fig. 3a).

In the subgroup of 200 patients who underwent mpMRI, the combination of mpMRI with urinary markers (mpMRI+Urine test) resulted in an AUC of 0.868, and the addition of both SOC and urinary markers to mpMRI (SOC + mpMRI + Urine test) further improved the AUC to 0.875. Both models outperformed mpMRI alone, with statistically significant differences (p < 0.0001) (Fig. 3b).

To assess the clinical utility of the predictive models, DCA was performed across a range of threshold probabilities.

**Fig. 4:**
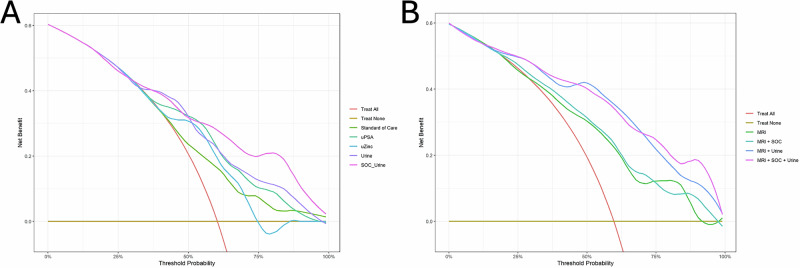
Decision curve analysis comparign the net clinical benefit of multiple predictive models. **A** The models evaluated include the Standard of Care (green), urinary biomarkers uPSA (teal) and uZinc (blue), a composite urinary model of uPSA intensity + uZinc intensity (Urine, purple), and a fully integrated clinical-urinary model (SOC_Urine, pink). Reference strategies of treating all patients (red) and treating none (yellow) are also shown. **B** The models include PIRADS alone (MRI, green), PIRADS with standard of care variables (MRI + SOC, turquoise), PIRADS with urinary PSA and Zinc markers (MRI + Urine, blue), and the fully integrated model combining MRI, clinical parameters, and urinary markers (MRI + SOC + Urine, pink). Reference strategies of treating all patients (red) and treating none (yellow) are also shown.

The first DCA revealed that the SOC + Urine model offered the highest net benefit across a broad range of threshold probabilities, consistently outperforming other models in balancing true positives and minimizing false positives. The Urine model also showed strong performance, particularly between 30% and 50% thresholds, suggesting these biomarkers capture relevant biological signals beyond standard clinical parameters. In contrast, the SOC model demonstrated limited net benefit, especially at higher thresholds, highlighting the added value of biomarker-enhanced approaches. (Fig. 4A).

The second DCA (Fig. 4B) focuses on patients who underwent mpMRI. The MRI model shows limited net benefit, performing similarly to the “Treat All” strategy at low thresholds and declining beyond 50–60%. MRI + SOC yields only marginal improvement. In contrast, MRI + Urine enhances net benefit, especially between 30% and 70%. The best performance is achieved by the fully integrated model, MRI + SOC + Urine, which consistently shows the highest net benefit across all thresholds, highlighting the value of combining imaging, clinical, and molecular data.

Among patients with ISUP4–5 tumors, 97% had a positive Urine test, compared to 87% in ISUP3 and 78% in ISUP1–2. Notably, 48% of healthy individuals had a negative test, showing no detectable markers (Supplementary Table 3).

Out of all the enrolled subjects, 50 met the inclusion criteria for algorithm-based urine testing. Among the eligible patients, 29 (58%) had a positive Urine test, and a biopsy was recommended. Of these, 12 (41%) were diagnosed with PCa, while 17 (59%) had negative biopsy results. The remaining 21 patients (42%) had a negative test and would have been directed to clinical follow-up. Within this group, 3 individuals (14%) were found to have PCa, while 18 (86%) had negative biopsies.

Notably, 51.4% of healthy subjects tested negative, suggesting a potential reduction of approximately half in unnecessary biopsies

Importantly, none of the cancers identified in the follow-up group had an ISUP grade higher than 2, indicating the absence of high-grade disease.

## Discussion

PCa is among the most common malignancies in men, yet no screening strategy has shown clear benefit so far [8–10]. Despite continuous updates to clinical guidelines, PCa diagnosis can be challenging due to the limited accuracy of the currently available diagnostic tools, starting from PSA [7, 12].

To address these limitations, several blood, urine and tissue-based tests have been developed [18–21].

Genetic tests, while informative for tumor biology, are limited in diagnostic use due to the multifocal and genetically heterogeneous nature of PCa. A single molecular signature often fails to represent the full disease spectrum, even within the same patient.

Similarly, while studies like the PICTURE trial have identified tissue biomarkers associated with cancer progression, their reliance on biopsy samples restricts their use to post-diagnosis settings [31].

Blood seems a more appropriate source of cancer-related biomarkers. The 4Kscore test is a blood-based assay that estimates the risk of high-grade PCa (Gleason ≥ 7) by combining four kallikrein markers—total PSA, free PSA, intact PSA, and hK2—with clinical variables like age, DRE findings, and biopsy history [32]. This test, though useful in biopsy decision-making, is limited by its dependence on PSA and centralized lab processing, reducing its specificity and general screening utility.

Urine represents an ideal medium for biomarker testing due to its non-invasive collection, high volume availability, and stable protein composition.

The ExoDx Prostate (EPI) test is a non-invasive urine test that helps identify men at low risk for high-grade prostate cancer, potentially reducing unnecessary biopsies [33]. However, its accuracy varies across populations and may miss aggressive cancers. Limited accessibility due to centralized lab processing, specialized infrastructure, and high costs further restricts its widespread use.

Our approach differs from many biomarker strategies that rely on complex molecular profiling or invasive sampling. By measuring two molecules, PSA and zinc, naturally produced by a healthy prostate, we capture functional changes that may indicate neoplastic transformation. This makes the test biologically intuitive and potentially universal, as it reflects a loss of normal prostate activity regardless of tumor genetics.

In recent years, numerous prostate cancer risk calculators (RCs) have emerged, combining clinical variables to estimate individual risk and reduce unnecessary biopsies. However, their use in the general population is limited, as many lack external validation and are based on highly selected cohorts, raising concerns about generalizability [34]. The integration of reliable biomarkers, such as uPSA and uZinc, could significantly enhance the accuracy and clinical utility of RCs, especially in the early screening phase.

In previous work, uPSA levels resulted significantly lower in PCa patients and inversely correlated with disease aggressiveness [25]. Similarly, uZinc was reduced in patients with ISUP ≥ 3 cancers, suggesting an association with more aggressive disease [24].

When combined, uPSA and uZinc enhanced diagnostic accuracy and showed stronger correlation with PCa severity [26].

Based on these premises, the results of the present study, aimed at developing a rapid Urine test combining uPSA and uZinc, deserve to be carefully analyzed and interpreted. First of all, it showed a strong correlation with standard methods used to determine PSA and zinc in urine confirming its reliability.

Levels of uPSA inversely correlated with ISUP and also reflected clinical stage and progression risk, while uZinc showed similar but weaker trends (Supplementary Table 1) confirming previous evidence [24–26].

Moreover, the proposed algorithm (Supplementary Fig. 2) may guide indication to prostate biopsy, particularly in ambiguous cases. In fact, when looking the 2025 EAU Guidelines [30], the indication for biopsy in PIRADS 3 cases with very low risk of PCa was reconsidered. In patients with similar characteristics, the availability of a diagnostic tool able to stratify ones deserving further investigation by one’s worthy of follow-up could optimize the diagnostic pathway, avoiding unnecessary procedures. Therefore, when looking at the algorithm performance on the cohort of this study, 86% of men candidates to prostate biopsy with a Urine test negative were indeed healthy, suggesting that 51% (18/35) of biopsies in healthy individuals could be avoided, at the cost of missing only 3 PCa cases. It has to be underlined that none of the missed cases had an ISUP score above 2. This implies that delayed biopsies in low-risk patients (who would still be monitored) are unlikely to impact the detection of csPCa. At last, given that test intensity correlates with ISUP (Supplementary Table 2), it may serve as a triage tool to reduce unnecessary biopsies and prioritize csPCa, potentially optimizing oncological and functional outcomes.

The growing availability of diverse diagnostic tools for prostate cancer—including serum PSA, mpMRI, clinical risk factors, and urinary biomarkers—has spurred interest in artificial intelligence (AI) approaches to integrate these data sources into clinically actionable models.

Our uPSA/uZinc test, with its semi-quantitative scoring system, lends itself to inclusion in AI models capable of learning from heterogeneous input modalities. Further prospective studies are needed to validate AI-driven diagnostic frameworks incorporating our rapid urine test in multicenter and real-world settings.

Concerning the potential applicability of the rapid urine test in clinical practice, in parallel with its diagnostic accuracy, also cost-effectiveness is another relevant factor to be considered. With growing pressure to reduce healthcare expenses, particularly in industrialized and resource-limited settings [27, 35], a simple and affordable test becomes highly valuable. Urine samples are easy to collect and interpret, requiring no specialized staff or equipment. If scaled for industrial production, this test could dramatically lower diagnostic costs compared to current laboratory-based biomarker tests as well as imaging tools like mpMRI, making PCa screening potentially more sustainable and accessible [36, 37].

Despite its advantages, the study has limitations. Not all patients underwent mpMRI and all were pre-selected for biopsy, limiting generalizability. Moreover, the relatively high overall cancer detection rate (60%) may reflect a degree of selection bias, potentially influenced by exclusion of patients with prior negative biopsies within 6-month. Broader validation is needed, and a multicenter study is planned. The test’s integration with mpMRI should be explored, as combining biomarkers and imaging may enhance accuracy and reduce unnecessary procedures.

Future research should assess whether urinary testing can streamline diagnostics by making the process faster and less MRI-dependent.

Moreover, although its use for the diagnosis of PCa has the potential to positively impact the economic burden on the healthcare system, no specific cost analysis or cost-saving evaluation has been conducted at this stage, as it falls outside the scope of the present study. Future research with an economic focus will specifically address these aspects.

In conclusion, this urinary test showed strong potential to optimize csPCa diagnosis by reducing unnecessary biopsies, guiding clinical decision making, and improving patient outcomes. Its simplicity, cost-effectiveness, and ability to prioritize based on disease aggressiveness could make it a valuable tool in the diagnostic path of PCa.

## Supplementary information

SUPPLEMENTARY MATERIAL

## Data Availability

The datasets analyzed during the current study are available from the corresponding author on reasonable request.
