# Supplementary material for: A rapid urinary test for combining PSA and zinc to enhance prostate cancer diagnosis: results from a prospective study
Source: Prostate Cancer Prostatic Dis. 2025 Dec 8;29(2):385–91. doi: 10.1038/s41391-025-01030-2 (PMC13190268; doi:10.1038/s41391-025-01030-2)

Supplementary Table 1. correlation matrix between clinical characteristics and biomarkers

**ISUP**		0,95<br>p<0,0001	0,811<br>p<0,0001	0,428<br>p<0,0001	0,346<br>p<0,0001	0,291<br>p<0,0001
**D'Amico**	0,95<br>p<0,0001		0,826<br>p<0,0001	0,377<br>p<0,0001	0,332<br>p<0,0001	0,232<br>0,0003
**Clinical Stage**	0,811<br>p<0,0001	0,826<br>p<0,0001		0,43<br>p<0,0001	0,316<br>p<0,0001	0,186<br>0,0038
**uPSA**	0,428<br>p<0,0001	0,377<br>p<0,0001	0,43<br>p<0,0001		0,427<br>p<0,0001	0,09<br>0,1666
**uZinco**	0,346<br>p<0,0001	0,332<br>p<0,0001	0,316<br>p<0,0001	0,427<br>p<0,0001		-0,014<br>p=0,8285
**blood PSA**	0,291<br>p<0,0001	0,232<br>p=0,0003	0,186<br>p=0,0038	0,09<br>p=0,1666	-0,014<br>p=0,8285	
	**ISUP**	**D'amico**	**Clinical Stage**	**uPSA**	**uZinco**	**blood PSA**

(Pearson correlation coefficient; ISUP: International Society of Uro-Pathology; uPSA: urine prostate specific antigen; uZinc: urinary Zinc)

Supplementary Table 2: ORs for SOC, MRI, Urine models and combinations

Model	OR	95% CI
**DRE**	1,6348	0,6852 to 3,9001
**PSA tot**	1,0277	0,9539 to 1,1074
**Age**	1,0419	0,9896 to 1,0969
**uPSA**	2,1914	1,5250 to 3,1490
**uZinc**	1,4107	1,0741 to 1,8527
**PIRADS**	3,5657	2,2083 to 5,7573

(OR: odd ratio; CI: confidence interval; DRE: digital rectal examination; PSA: prostate specific antigen; uPSA: urinary PSA; uZinc: urinary Zinc; PIRADS: Prostate Imaging–Reporting and Data System)

Supplementary Table 3: Distribution of Urine Scores in ISUP groups

Urine Score	Healthy subjects n (%)	ISUP 1-2 n (%)	ISUP 3 n (%)	ISUP 4-5 n (%)
**2**	13 (14)	27 (39)	18 (47)	25 (64)
**1**	37 (39)	27 (39)	15 (39)	13 (33)
**0**	46 (48)	15 (22)	5 (13)	1 (3)

(ISUP: International Society of Uro-Pathology)

Supplementary Figure 1: ROC curves of uPSA and uZinc thresholds

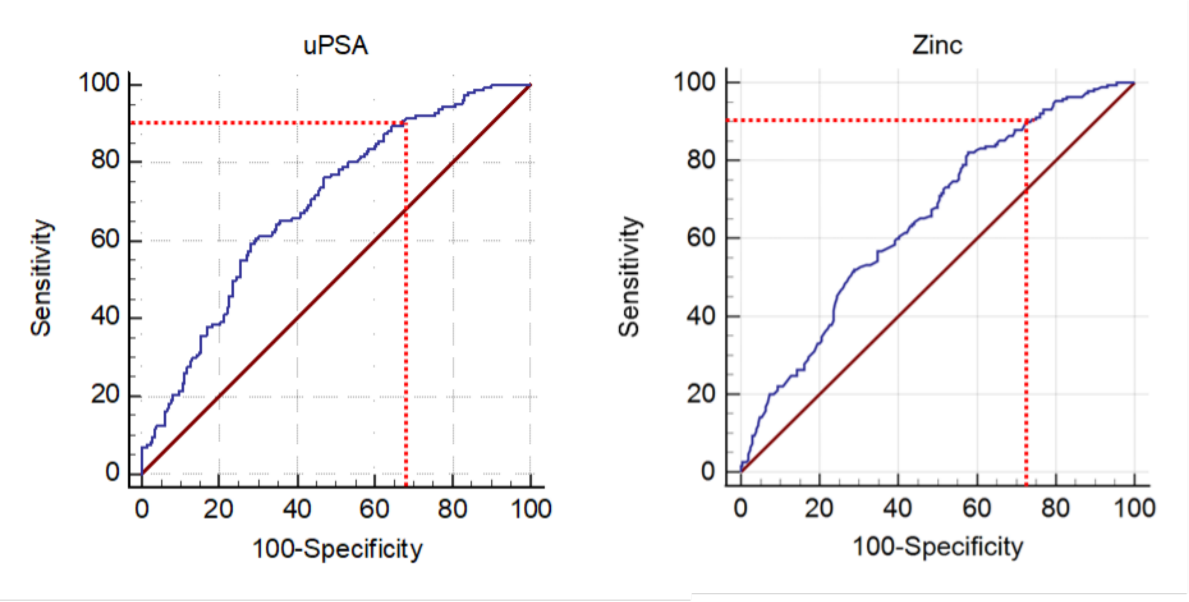

Supplementary Figure 2: decision-making algorithm to indicate prostate biopsy based on PSA, mpMRI and DRE

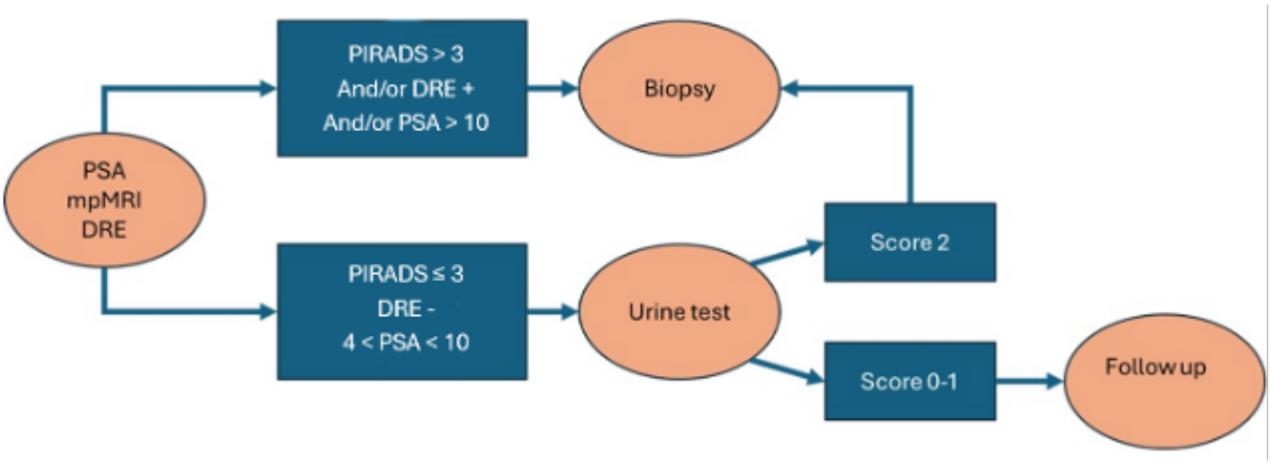

Supplementary Figure 3: Comparison between rapid test and reference methods for uPSA (A) and uZinc (B)

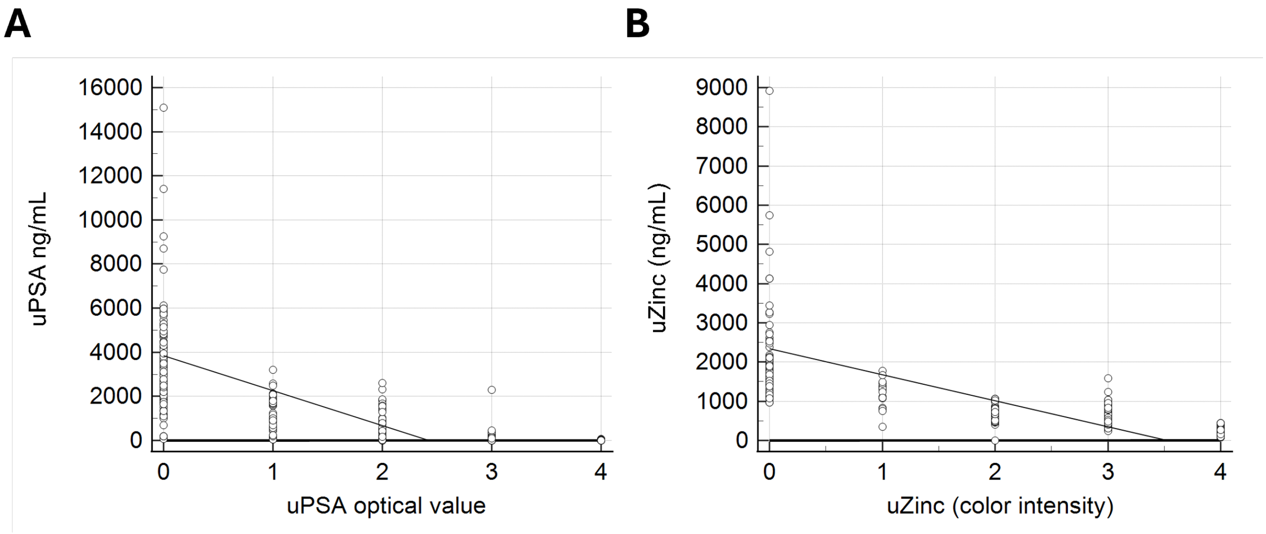

Supplement: Supplementary file 1 — SUPPLEMENTARY MATERIAL [file 41391_2025_1030_MOESM1_ESM.pdf]
